# The Prevalence and Impact of Model Violations in Phylogenetic Analysis

**DOI:** 10.1093/gbe/evz193

**Published:** 2019-09-19

**Authors:** Suha Naser-Khdour, Bui Quang Minh, Wenqi Zhang, Eric A Stone, Robert Lanfear

**Affiliations:** 1 Department of Ecology and Evolution, Research School of Biology, Australian National University, Canberra, Australian Capital Territory, Australia; 2 Research School of Computer Science, Australian National University, Canberra, Australian Capital Territory, Australia

**Keywords:** model violations, phylogenetic inference, test of symmetry, systematic bias

## Abstract

In phylogenetic inference, we commonly use models of substitution which assume that sequence evolution is stationary, reversible, and homogeneous (SRH). Although the use of such models is often criticized, the extent of SRH violations and their effects on phylogenetic inference of tree topologies and edge lengths are not well understood. Here, we introduce and apply the maximal matched-pairs tests of homogeneity to assess the scale and impact of SRH model violations on 3,572 partitions from 35 published phylogenetic data sets. We show that roughly one-quarter of all the partitions we analyzed (23.5%) reject the SRH assumptions, and that for 25% of data sets, tree topologies inferred from all partitions differ significantly from topologies inferred using the subset of partitions that do not reject the SRH assumptions. This proportion increases when comparing trees inferred using the subset of partitions that rejects the SRH assumptions, to those inferred from partitions that do not reject the SRH assumptions. These results suggest that the extent and effects of model violation in phylogenetics may be substantial. They highlight the importance of testing for model violations and possibly excluding partitions that violate models prior to tree reconstruction. Our results also suggest that further effort in developing models that do not require SRH assumptions could lead to large improvements in the accuracy of phylogenomic inference. The scripts necessary to perform the analysis are available in https://github.com/roblanf/SRHtests, and the new tests we describe are available as a new option in IQ-TREE (http://www.iqtree.org).

## Introduction

Phylogenetics is an essential tool for inferring evolutionary relationships between individuals, species, genes, and genomes. Moreover, phylogenetic trees form the basis of a huge range of other inferences in evolutionary biology, from gene function prediction to drug development and forensics (Eisen 1998; Farrell et al. 2000; Mäser et al. 2001; Gardner et al. 2002; Yao et al. 2003, 2004; Grenfell et al. 2004; Salipante and Horwitz 2006; Gray et al. 2009; Brady and Salzberg 2011; Dunn et al. 2011).

Most phylogenetic studies use models of sequence evolution which assume that the evolutionary process follows stationary, reversible, and homogeneous (SRH) conditions. Stationarity implies that the marginal frequencies of the nucleotides or amino acids are constant over time, reversibility implies that the evolutionary process is stationary and undirected (substitution rates between nucleotides or amino acids are equal in both directions), and homogeneity implies that the instantaneous substitution rates are constant along the tree or over an edge (Felsenstein 2004; Yang and Rannala 2012; Jermiin et al. 2017). However, these simplifying assumptions are often violated by real data (Foster and Hickey 1999; Tarrío et al. 2001; Paton et al. 2002; Goremykin and Hellwig 2005; Murray et al. 2005; Bourlat et al. 2006; Hyman et al. 2007; Sheffield et al. 2009; Nesnidal et al. 2010; Nabholz et al. 2011; Martijn et al. 2018). Such model violation may lead to systematic error that, unlike stochastic error, cannot be remedied simply by increasing the size of a data set (Felsenstein 2004; Ho and Jermiin 2004; Jermiin et al. 2004; Philippe et al. 2005; Sullivan and Joyce 2005; Kumar et al. 2012; Brown and Thomson 2017; Duchene et al. 2017). As phylogenetic data sets are steadily growing in terms of taxonomic and site sampling, it is vital that we develop and employ methods to measure and understand the extent to which systematic error affects phylogenetic inference (systematic bias), and explore ways of mitigating this systematic bias in empirical studies.

One approach to accommodate data that have evolved under non-SRH conditions is to employ models that relax the SRH assumptions. A number of non-SRH models have been implemented in a variety of software packages (Foster 2004; Lartillot and Philippe 2004; Blanquart and Lartillot 2006; Boussau and Gouy 2006; Jayaswal et al. 2007, 2011, 2014; Knight et al. 2007; Dutheil and Boussau 2008; Sumner et al. 2012; Zou et al. 2012; Groussin et al. 2013; Nguyen et al. 2015; Woodhams et al. 2015). However, such models remain infrequently used as searching for optimal phylogenetic trees under these models is computationally demanding (Betancur-r et al. 2013) and the implementations are often not easy to use. As a result, the vast majority of empirical phylogenetic inferences rely on models that assume sequences have evolved under SRH conditions, such as the general time reversible family of models implemented in many of the most widely used phylogenetics software packages (Swofford 2001; Drummond and Rambaut 2007; Guindon et al. 2010; Ronquist et al. 2012; Bazinet et al. 2014; Bouckaert et al. 2014; Stamatakis 2014; Nguyen et al. 2015; Höhna et al. 2016).

Another approach to accounting for data that may have evolved under non-SRH conditions is to test for model violations prior to tree reconstruction. Here, one first screens data sets or parts of data sets, and reconstructs trees exclusively from data that do not reject SRH conditions. A number of methods have been proposed to test for violation of SRH conditions in aligned sequences prior to estimating trees (Bowker 1948; Stuart 1955; Rzhetsky and Nei 1995; Kumar and Gadagkar 2001; Weiss and von Haeseler 2003; Ababneh et al. 2006; Ho et al. 2006), and there are also a posteriori tests for absolute model adequacy which are employed after trees have been estimated (Goldman 1993; Bollback 2002; Brown and ElDabaje 2009; Brown 2014; Duchene et al. 2017; Brown and Thomson 2018).

Allowing the data to reject the model when the assumptions of the model are violated is an important approach to reducing systematic bias in phylogenetic inference (Philippe et al. 2005; Brown 2014). Knowing in advance which sequences and loci are inconsistent with the SRH assumptions will allow us to choose more complex models or to omit some of these sequences and loci from downstream analyses (Kumar and Gadagkar 2001). The need for methods that assess the evolutionary process prior to phylogenetic inference becomes more important as the number of sequences and sites per data set increases, because systematic bias has an increasing effect on inferences from larger phylogenetic data sets (Ho and Jermiin 2004; Jermiin et al. 2004; Phillips et al. 2004; Delsuc et al. 2005).

In this article, we evaluate the extent and effect of model violation due to non-SRH evolution using 35 empirical data sets with a total of 3,572 partitions. We determine if the SRH assumptions are violated by extending and applying the matched-pairs tests of homogeneity (Jermiin et al. 2017) to each partition. We then compare the phylogenetic trees for each data set estimated from all of the partitions, the partitions that reject the SRH assumptions, and the partitions that do not reject the SRH assumptions, in order to evaluate the effect violating SRH conditions on phylogenetic inference. Our results suggest that violating SRH assumptions can have substantial impacts on phylogenetic inference.

## Materials and Methods

### Empirical Data Sets

In order to assess the impact of model violation in phylogenetics, we first gathered a representative sample of 35 partitioned empirical data sets that had been used for phylogenetic analysis in recent studies (table 1). Within the constraints of selecting data that were publicly available and suitably annotated, that is, such that all loci and all codon positions within protein-coding loci could be identified, we selected the data sets to provide as representative a sample as possible of the data types, taxa, and genomic regions most commonly used to infer bifurcating phylogenetic trees from concatenated alignments. These data sets include nucleotide sequences from nuclear, mitochondrial, plastid, and virus genomes, and include protein-coding DNA, introns, intergenic spacers, tRNA, rRNA, and ultraconserved elements. The number of taxa and sites in these data sets range from 27 to 355 and from 699 to 1,079,052, respectively. The clades represented in these data sets include animals, plants, and viruses. We partitioned all data sets to the maximum possible extent based on the biological properties of the data, that is, we divided every locus and every codon position within each protein-coding locus into a separate partition. All partitioning information is available at the github repository (https://github.com/roblanf/SRHtests/tree/master/datasets), and the full details of every data set are provided in table 1 and in supplementary extended table 5, Supplementary Material online.

**Table 1:** Number of Taxa, Number of Sites, Clade, and Study Reference for Each Data Set That Have Been Used in This Study

Data Set	Study References	Data Set References	Clade	Taxa	Sites
Anderson_2013	Anderson et al. (2014)	Anderson et al. (2013)	Loliginids	145	3,037
Bergsten_2013	Bergsten et al. (2013)	Bergsten et al. (2013)	Dytiscidae	38	2,111
Broughton_2013	Broughton et al. (2013)	Broughton et al. (2013)	Osteichthyes	61	19,997
Brown_2012	Brown et al. (2012)	Brown et al. (2012)	Ptychozoon	41	1,665
Cannon_2016a	Cannon et al. (2016)	Cannon et al. (2016)	Metazoa	78	89,792
Cognato_2001	Cognato and Vogler (2001)	Cognato and Vogler (2001)	Coleoptera: Scolytinae	44	1,897
Day_2013	Day et al. (2013)	Day et al. (2013)	Synodontis	152	3,586
Devitt_2013	Devitt et al. (2013)	Devitt et al. (2013)	Ensatina eschscholtzii klauberi	69	823
Dornburg_2012	Dornburg et al. (2012)	Dornburg et al. (2012)	Teleostei: Beryciformes: Holocentridae	44	5,919
Faircloth_2013	Faircloth et al. (2013)	Faircloth et al. (2013)	Actinopterygii	27	149,366
Fong_2012	Fong et al. (2012)	Fong et al. (2012)	Vertebrata	110	25,919
Horn_2014	Horn et al. (2014)	Horn et al. (2014)	Euphorbia	197	11,587
Kawahara_2013	Kawahara and Rubinoff (2013)	Kawahara and Rubinoff (2013)	Hyposmocoma	70	2,238
Lartillot_2012	Lartillot and Delsuc (2012)	Lartillot and Delsuc (2012)	Eutheria	78	15,117
McCormack_2013	McCormack et al. (2013)	McCormack et al. (2013)	Neoaves	33	1,079,052
Moyle_2016	Moyle et al. (2016)	Moyle et al. (2016)	Oscines	106	375,172
Murray_2013	Murray et al. (2013)	Murray et al. (2013)	Eucharitidae	237	3,111
Oaks_2011	Oaks (2011)	Oaks (2011)	Crocodylia	79	7,282
Rightmyer_2013	Rightmyer et al. (2013)	Rightmyer et al. (2013)	Hymenoptera: Megachilidae	94	3,692
Sauquet_2011	Sauquet et al. (2012)	Sauquet et al. (2011)	Nothofagus	51	5,444
Seago_2011	Seago et al. (2011)	Seago et al. (2011)	Coccinellidae	97	2,253
Sharanowski_2011	Sharanowski et al. (2011)	Sharanowski et al. (2011)	Braconidae	139	3,982
Siler_2013	Siler et al. (2013)	Siler et al. (2013)	Lycodon	61	2,697
Tolley_2013	Tolley et al. (2013)	Tolley et al. (2013)	Chamaeleonidae	203	5,054
Unmack_2013	Unmack et al. (2013)	Unmack et al. (2013)	Melanotaeniidae	139	6,827
Wainwright_2012	Wainwright et al. (2012)	Wainwright et al. (2012)	Acanthomorpha	188	8,439
Wood_2012	Wood et al. (2013)	Wood et al. (2012)	Archaeidae	37	5,185
Worobey_2014a	Worobey et al. (2014)	Worobey et al. (2014)	Influenzavirus A	146	3,432
Worobey_2014b	Worobey et al. (2014)	Worobey et al. (2014)	Influenzavirus A	327	759
Worobey_2014c	Worobey et al. (2014)	Worobey et al. (2014)	Influenzavirus A	92	1,416
Worobey_2014d	Worobey et al. (2014)	Worobey et al. (2014)	Influenzavirus A	355	1,497
Worobey_2014e	Worobey et al. (2014)	Worobey et al. (2014)	Influenzavirus A	340	699
Worobey_2014f	Worobey et al. (2014)	Worobey et al. (2014)	Influenzavirus A	332	2,151
Worobey_2014g	Worobey et al. (2014)	Worobey et al. (2014)	Influenzavirus A	326	2,274
Worobey_2014h	Worobey et al. (2014)	Worobey et al. (2014)	Influenzavirus A	351	2,280

### Workflow Summary

**Fig. 1.**
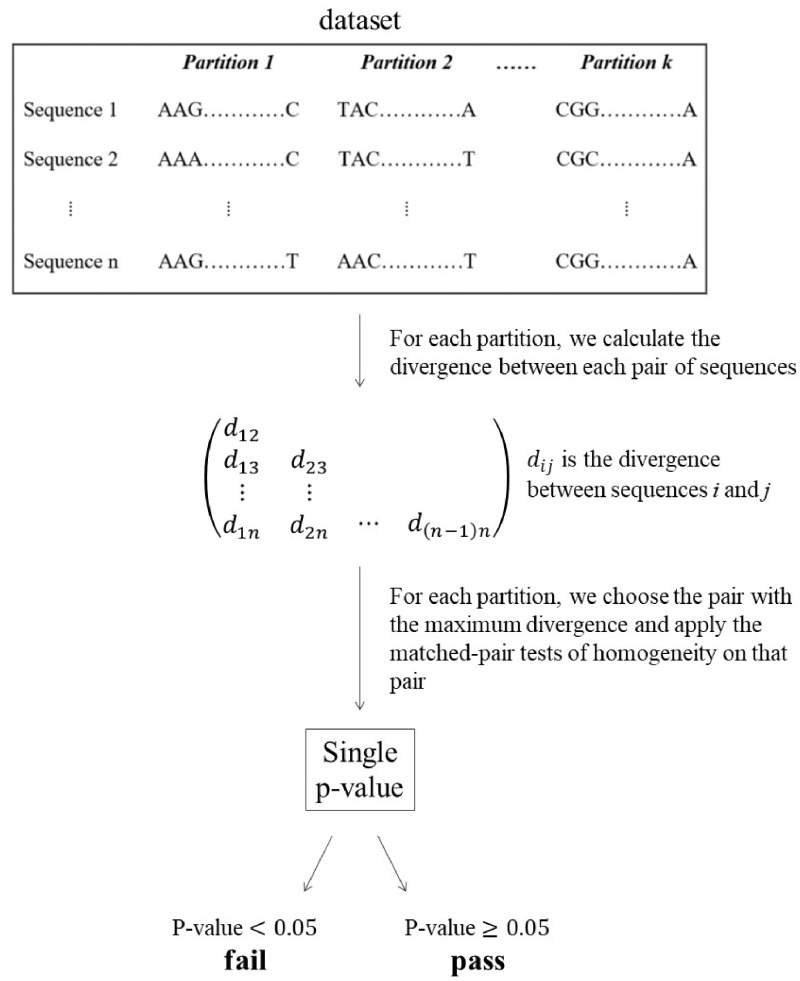
—Flow chart of methodology. For each partition in the alignment, we choose the pair of sequences with the maximum divergence and apply the matched-pairs tests of homogeneity on that pair.

Figure 1 outlines the workflow. For each partition in each data set, we used a new approach based on the three matched-pairs tests of homogeneity to ask whether the evolution of the aligned sequences in the partition rejects the SRH assumptions. The three matched-pairs tests of homogeneity, described in more detail below, test three slightly different assumptions about the historical process that generated each aligned pair of sequences in a given partition. A significant result from any test suggests that the nature of the evolutionary process required to explain the aligned sequences violates at least one of the three SRH conditions (Jermiin et al. 2017). For each test, we classify each partition as *pass* if the result of the test is nonsignificant or *fail* if the result of the test is significant. We then denote the original data set as *D*_all_, while the concatenation of *pass* partitions is denoted *D*_pass_ and the concatenation of *fail* partitions as *D*_fail_ (fig. 1).

To investigate the impact of model violation on phylogenetic inference, we infer and compare three phylogenetic trees, *T*_all_, *T*_pass_, and *T*_fail_, estimated from *D*_all_, *D*_pass_, and *D*_fail_, respectively.

### Matched-Pairs Tests of Homogeneity

The three matched-pairs tests of homogeneity that are applied to pairs of sequences are: the MPTS (matched-pairs test of symmetry), MPTMS (matched-pairs test of marginal symmetry), and MPTIS (matched-pairs test of internal symmetry). The statistics are computed on an *m*-by-*m* (*m* is 4 for nucleotides and 20 for amino acids) divergence matrix D with elements $d_{\mathrm{ij}}$, where $d_{\mathrm{ij}}$ is the number of alignment sites having nucleotide (or amino acid) i in the first sequence and nucleotide (or amino acid) j in the second sequence.

The MPTS tests the symmetry of D by computing the Bowker’s (1948) test statistic as the χ^2^ distance between D and its transpose:

$$S_{B}^{2}=\sum_{1\leq i<\;j\leq m}\frac{{\left(d_{\mathrm{ij}}\;-\;d_{\mathrm{ji}}\right)}^{2}}{\left(d_{\mathrm{ij}}\;+\;d_{\mathrm{ji}}\right)},$$

where $d_{\mathrm{ij}}+d_{\mathrm{ji}}>0$. A *P* value is then obtained by a χ^2^ test with f degrees of freedom, where f is the number of $\left(i,j\right)$ pairs for which $d_{\mathrm{ij}}+d_{\mathrm{ji}}>0$. A small *P* value (e.g., <0.05) indicates that the assumption of symmetry is rejected at that significance level, suggesting that evolution is nonstationary, nonhomogeneous, or both (Jermiin et al. 2017).

The MPTMS tests the equality of nucleotide or amino acid composition between two sequences. To do so, MPTMS computes the Stuart’s test statistic $S_{S}^{2}=\;u^{T}V^{-1}u$ using the difference between nucleotide or amino acid frequencies of two sequences, u, and its variance–covariance matrix, V. In detail, u is given by $u^{T}=(d_{1•}-d_{•1},\;d_{2•}-d_{•2},\ldots ,d_{k•}-d_{•k})$ where $d_{i•}$ is the sum of $d_{\mathrm{ij}}$ over *j*, $d_{•j}$ is the sum of $d_{\mathrm{ij}}$ over *i*, and, *k *=* m*−1. V, the estimated variance–covariance matrix of *u* under the assumption of marginal symmetry, is defined elementwise by:

$$v_{\mathrm{ij}}=\left\{\begin{array}{c} d_{i•}+\;d_{•i}-2d_{\mathrm{ii}},\;i=j\;\\ -\left(d_{\mathrm{ij}}+d_{\mathrm{ji}}\right),\;i\neq j\; \end{array}\right..$$

A *P* value is obtained by a χ^2^ test with *m*−1 degrees of freedom. A small *P* value (<0.05) indicates that the stationarity assumption is rejected. Note that when V is not invertible, the Stuart’s statistic $S_{S}^{2}$ is ill-defined and the MPTMS is not applicable.

The MPTIS uses the test statistic as the difference between Bowker’s and Stuart’s statistic:

$S_{I}^{2}=\;S_{B}^{2}-S_{S}^{2}$. $S_{I}^{2}$ is χ^2^ distributed with f-m+1 degrees of freedom. A small *P* value (<0.05) indicates that the homogeneity assumption is rejected.

The MPTS, MPTMS, and MPTIS test different aspects of the symmetry with which differences accumulate between pairs of sequences due to the substitution process. The MPTS is a comprehensive and sufficient test to determine whether the data comply with the SRH assumptions (Jermiin et al. 2017), but it cannot provide any information about the source of this violation. Some information on the underlying source of model violation may be obtained by performing the other two tests of symmetry: the MPTMS and the MPTIS. If the violation of the SRH assumptions stems from differences in base composition between the sequences, this should affect the marginal symmetry of the sequence pair, which can in principle be detected by the MPTMS. If the violation of the SRH assumptions stems from changes in the relative substitution rates over time, this should affect the internal symmetry of the sequence pair, which can in principle be detected by the MPTIS. However, even after performing all three tests, it is difficult to ascertain which of the three SRH assumptions is violated during the evolutionary process because the relationships between the SRH conditions and the three matched-pair tests is neither bijective nor injective, that is, there is not a one-to-one correspondence between the three tests and violation of the three SRH conditions (Jermiin et al. 2017).

The three matched-pairs tests of homogeneity are appropriate to test for SRH assumptions as they consider the alignment on a site-by-site basis. The basic intuition that underlies these tests is that two sequences diverging under SRH conditions should accumulate differences symmetrically (e.g., both sequences are equally likely to accumulate at a C to T change at a site in which both originally shared a C). This symmetry of accumulation is reflected by symmetries in the resulting difference matrix, violations of which can be assessed statistically. However, these tests were designed to ask whether any single pair of sequences rejects the SRH conditions (Jermiin et al. 2017). To ask whether a given partition rejects SRH conditions, we developed an approach to extend the matched-pairs tests of homogeneity to accommodate data sets with more than two sequences.

### Maximum Symmetry Test

In order to determine whether a given multiple sequence alignment rejects SRH conditions, we consider only the pair of taxa with the maximum divergence. In order to find the maximum divergent pair, we sum the off-diagonal elements of the divergence matrix and divide by the sum of all elements. We then randomly choose one pair from all the pairs with the maximum divergence score (if there is more than one pair). By using the most divergent sequence pair, we maximize our power to detect model violations without a priori knowledge of the underlying tree topology and the dependencies that it induces in the data. For the maximum divergent pair, we then apply the matched-pair tests of homogeneity and calculate their χ^2^*P* values. If the obtained *P* value is <0.05, then we consider that the null hypothesis of SRH evolution is rejected for the corresponding partition and we add it to the *D*_fail_ data set. Otherwise, we add it to the *D*_pass_ data set. We denote our applications of the MPTS, MPTMS, and MPTIS based on the $d_{\max}\;\mathrm{Pair}$ as MaxSymTest, MaxSymTest_mar_, and MaxSymTest_int_, respectively.

### Phylogenetic Inference

We used IQ-TREE (Nguyen et al. 2015) to infer up to seven phylogenetic trees for every data set: *T*_all_ (all partitions from the original data set; *D*_all_); and *T*_pass_ and *T*_fail_ based on the *D*_pass_ and *D*_fail_ data sets from each of the three tests (MaxSymTest, MaxSymTest_mar_, MaxSymTest_int_), provided that there was at least one partition in each category. We ran IQ-TREE using the default settings with the best-fit fully partitioned model (Chernomor et al. 2016), which allows each partition to have its own evolutionary model and edge-linked rate determined by ModelFinder (Kalyaanamoorthy et al. 2017) followed 1,000 ultrafast bootstrap replicates (Hoang et al. 2018).

### Distance between Trees

For each of the three tests (MPTS, MPTMS, MPTIS) we calculated the Normalized Path-Difference (NPD) and quartet distance (QD) (Steel and Penny 1993; Sand et al. 2014) between all three possible pairs of trees (*T*_all_ vs. *T*_pass_; *T*_all_ vs. *T*_fail_; and *T*_pass_ vs. *T*_fail_), as long as *D*_pass_ and *D*_fail_ were nonempty and so *T*_pass_ and *T*_fail_ had been estimated. The path-difference metric (PD) is defined as the Euclidean distance between pairs of taxa (Steel and Penny 1993; Mir and Russello 2010). In this study, because we are interested only in differences between topologies, we use the variant of the PD metric that ignores branch lengths. In order to compare path distances between trees with different number of taxa, we normalized PD (to obtain NPD) by the mean of a null distribution of PDs generated from 10K random pairs of trees with the same number of taxa (Bogdanowicz et al. 2012). Thus, an NPD of 0 indicates an identical pair of trees, an NPD of 1 indicates that a pair of trees is as similar as a pair of randomly selected trees with the same number of taxa; and an NPD >1 indicates a pair of trees that are less similar than a randomly selected pair of trees with the same number of taxa. Since path differences are always nonnegative, the NPD is also guaranteed to be nonnegative.

The QD metric is defined as the fraction of quartets (subsets of four taxa) that induce different subtrees between the two trees being compared. QD ranges between 0 and 1, where 0 means that two trees are identical and 1 means that they do not share any quartet subtrees. Compared with PD, QD has the advantage that its distribution is less sensitive to the underlying distribution of tree topologies (Steel and Penny 1993).

### Tree Topology Tests

The NPD and the QD give us measures of the differences between pairs of trees, but they do not tell us whether the differences are phylogenetically significant in the three data sets (*D*_pass_, *D*_all_, and *D*_fail_) derived from a given test. For example, trees that differ due to stochastic error associated with small data sets may be very different, but such differences may not be statistically significant. To assess the significance of the differences between *T*_pass_, *T*_all_, and *T*_fail_, we used the weighted Shimodaira–Hasegawa (wSH) test (Shimodaira and Hasegawa 1999; Shimodaira 2002) implemented in IQ-TREE with 1,000 RELL replicates (Kishino et al. 1990). Given the alignment (*D*_pass_), the wSH test computes a *P* value for each tree, where a small *P* value (<0.05) implies that the corresponding tree has a significantly worse likelihood than the best tree in the set of *T*_pass_, *T*_all_, and *T*_fail_. We use *D*_pass_ for these tests because it is, by definition, the only data set that does not reject the underlying assumptions of the SH test. As such, we only compute sWH *P* values when *D*_pass_ is nonempty. Thus, we performed a wSH test for each of the three MaxSymTest variants: each of which asks whether *T*_all_ and/or *T*_fail_ can be rejected in favor of *T*_pass_.

### Correlation between Number of Substitutions and Model Violation

We hypothesized that partitions with more substitutions may be more likely to violate the SRH assumptions, since substitutions form the raw data for the matched-pairs tests of homogeneity. To assess this, we fitted a linear mixed-effects model for each of the three tests using the glmer function from the lme4 package in R (Bates et al. 2015). In this model, we treat each partition as a datapoint, the number of substitutions measured for that partition as a fixed effect, and the data set from which that partition was taken as a random effect. This allows us to estimate the extent to which the number of substitutions in a partition associates with whether a partition fails a given test of symmetry, after accounting for differences between the data sets. To calculate the *R*^2^ value, we use the r.squaredGLMM function from the MuMIn package in R (Barton 2009; Nakagawa and Schielzeth 2013).

### Software Implementation

We implemented a new option –symtest in IQ-TREE to perform the three MaxSymTest matched-pairs tests of symmetry. In addition, the option –symtest-remove-bad allows users to remove from the final analysis partitions that fail the MaxSymTest. One can change the removal criterion to MaxSymTest_mar_ or MaxSymTest_int_ via the –symtest-type MAR|INT option. In addition, the cutoff *P* value can be changed using the –symtest-pval NUM option, where the default value is 0.05.

### Reproducibility

The GitHub repository (https://github.com/roblanf/SRHtests) contains the raw data and Python and R scripts necessary to perform all analyses reported in this study.

## Results

### Violation of SRH Conditions Is Common across 35 Empirical Data Sets

Across all 3,572 partitions analyzed, 573 (16.0%) failed the MaxSymTest, 728 (20.4%) failed the MaxSymTest_mar_, and 312 (2.8%) failed the MaxSymTest_int_. In total, 840 (23.5%) of the partitions failed at least one test.

**Fig. 2.**
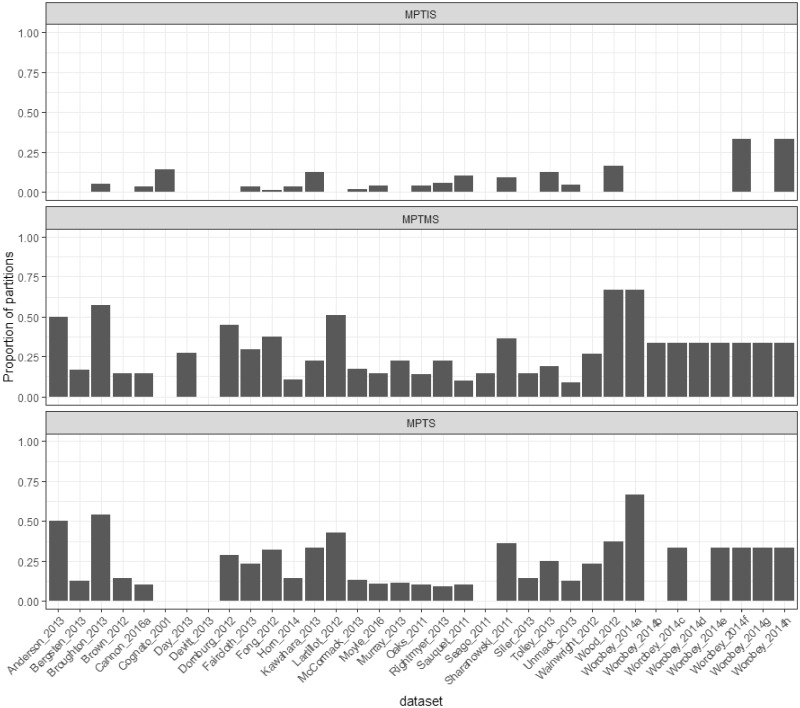
—The proportion of partitions that reject the null hypothesis of the MaxSymTest, MaxSymTest_mar_, and MaxSymTest_int_ (*P* value <0.05) in each data set.

The proportion of partitions failing each test varied substantially among data sets (fig. 2), but on an average, 21.8% of the partitions in each data set failed the MaxSymTest, 27.5% failed the MaxSymTest_mar_, and 5.1% failed the MaxSymTest_int_.

The fraction of failing partitions also varied with the genome type (e.g., mitochondrial, chloroplast, or nuclear) and context (e.g., protein-coding, UCE, tRNA) from which the partition was sequenced (table 2) although we note that a substantial proportion of the partitions from almost every category failed at least one of the tests (table 2).

**Table 2:** The Proportion of Partitions That Failed At Least One of the Three Tests—MaxSymTest, MaxSymTest_mar_, and MaxSymTest_int_

Type/Genome	Nuclear	Mitochondrial	Plastid	Virus
First codon positions	20.2%	27.6%	33.3%	25.0%
Second codon positions	21.0%	7.4%	0.0%	25.0%
Third codon positions	76.6%	44.8%	0.0%	75.0%
Other (e.g., intron)	27.8%	100.0%	0.0%	
rRNA	30.0%	25.0%		
UCE	22.5%			
tRNA		0.0%		

There were no clear differences in the substitution models that were selected for the partitions that pass or fail the tests (see supplementary extended tables 1–3, Supplementary Material online). However, we note that the two most-frequently selected substitution models (for 35% of the partitions) were relatively simple: K80 (Kimura 1980) and HKY (Hasegawa et al. 1985).

### Model Violation Has a Large Influence on Tree Topologies

Using both MaxSymTest and MaxSymTest_mar_, we compared each tree inferred from each data set (*T*_all_) to the corresponding trees estimated from the failed (*T*_fail_) and passed (*T*_pass_) partitions. Disturbingly, for each of the two tree distance metrics that we considered (NPD and QD), we find that the tree inferred from the original data set tended to be more similar to the tree estimated from the failed partitions (table 3 and supplementary extended table 4, Supplementary Material online). Furthermore, the mean NPD distance between *T*_pass_ and *T*_fail_ across all 35 data sets for the MaxSymTest was 0.69, that is, the two trees are 69% as dissimilar as random pairs of trees. This suggests that violations of SRH assumptions drive large changes in tree topologies.

**Table 3:** The Proportion of Data Sets That Have the Highest NPD Metric (and QD metric) between the Three Comparisons (All-fail, All-pass, Pass–fail) for MaxSymTest, MaxSymTest_mar_, and MaxSymTest_int_

	*T*_fail_	*T*_pass_
MaxSymTest		
*T*_all_	14.3% (4.8%)	4.8% (4.8%)
*T*_pass_	80.9% (90.4%)	
MaxSymTest_mar_		
*T*_all_	8.3% (0.0%)	8.3% (4.2%)
*T*_pass_	83.4% (95.8%)	
*MaxSymTest_int_*		
*T*_all_	28.6% (28.6%)	0.0% (0.0%)
*T*_pass_	71.4% (71.4%)	

The results of the wSH tests (table 4) confirm that the differences between trees that we observe tend to be statistically significant. For example, when using the MaxSymTest_mar_, *T*_pass_ is a significantly better description of the *D*_pass_ data than *T*_all_ in ∼37% of the data sets, and better than *T*_fail_ in ∼89% of the data sets.

**Table 4:** The Proportion of Data Sets That Have a Significant *P* Value in the Weighted SH Test When Using *D*_pass_ As the Input Alignment for the Test

	*T*_all_	*T*_fail_
MaxSymTest	25%	79%
MaxSymTest_mar_	37%	89%
MaxSymTest_int_	4%	28%

### The Number of Substitutions Explains Less than One-Third of the Variance in Passing or Failing the Tests of Symmetry

The number of substitutions in a partition explained 27.5% of the variation in whether or not a partition passed or failed the MaxSymTest (supplementary extended fig. 7, Supplementary Material online). This proportion is very similar for MaxSymTest_mar_ (24.4%) (supplementary extended fig. 8, Supplementary Material online), but is dramatically lower for the MaxSymTest_int_ (1.8%) (supplementary extended fig. 9, Supplementary Material online). Thus, although the number of substitutions in a partition is a highly significant (*P* < 2e-16) predictor of passing or failing any of the tests, that it explains only about a quarter of the variation suggests that other factors, such as underlying differences in the extent to which partitions violate the SRH assumptions, are driving the remaining ∼75% of the variation.

### Model Violation Due to Non-SRH Evolution Affects the Inferred Relationship between Even-Toed and Odd-Toed Ungulates in the Tree of Mammals

**Fig. 3.**
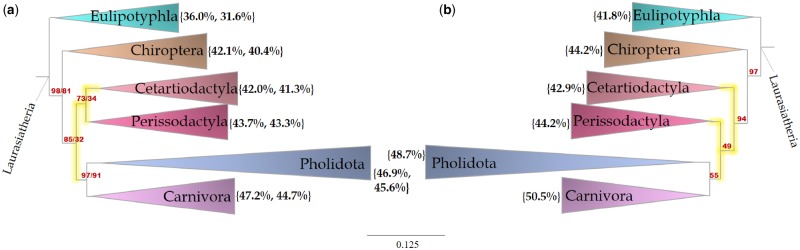
—Maximum-likelihood trees of mammalian relationships based on analysis of Lartillot 2012 data set. (*a*) The tree inferred from all 51 partitions and from the 29 partitions that passed the MaxSymTest. (*b*) The tree inferred from 22 partitions that failed the MaxSymTest. Red numbers at the internal branches indicate the bootstrap support values that are <100% under the best fitting model. Numbers in curly brackets show the GC content (in panel *a*, %GC and bootstrap support values are for *T*_all_ and *T*_pass_, respectively).

To examine the effects of model violation in more detail, we selected two data sets for more detailed consideration. Conflicting support for the placement of Xenacoelomorpha, the clade that contains Xenoturbella and Acoelomorpha, in the tree of life across different analyses has led to various hypotheses about the evolution of Bilateria (Cannon et al. 2016). In addition, the interordinal relationships in Laurasiatheria, especially the relationships between Fereuungulata (Perissodactyla, Cetartiodactyla, Carnivora, and Pholidota), in the tree of placental mammals is controversial (Cao et al. 1998; Zhou et al. 2012). It has been suggested that such inferences might be strongly affected by model violation and systematic error (Cao et al. 1998; Delsuc et al. 2005; Philippe et al. 2011; Tsagkogeorga et al. 2013). To assess whether data that pass or fail the MaxSymTest_mar_ show different signals regarding the evolution of the Bilateria and the superorder Laurasiatheria, we examined in more detail the *T*_all_, *T*_pass_, and *T*_fail_ trees from recent studies that explored the tree of placental mammals (Lartillot and Delsuc 2012) and the tree of all animals (Cannon et al. 2016). The mammals’ data set comprises 78 mammalian taxa, including 73 placental mammals with 51 partitions representing the first, second, and third codon positions of the 17 genes (Lartillot and Delsuc 2012). The tree reconstructed from all of the partitions (*T*_all_) and the tree reconstructed from the partitions that pass the MaxSymTest (*T*_pass_, 29 partitions) both show Perissodactyla (odd-toed ungulates) as a sister group to Cetartiodactyla (even-toed ungulates) (fig. 3*a* and supplementary extended figs. 4 and 5, Supplementary Material online). Even so, the bootstrap support for this branch is not high: 73% for *T*_all_ and 34% for *T*_pass_. On the other hand, the tree reconstructed from the data that fail the MaxSymTest (*T*_fail_, 22 partitions) shows Perissodactyla as the sister group to the clade that contains Carnivora + Pholidota with 49% bootstrap support (fig. 3*b* and supplementary extended fig. 6, Supplementary Material online).

The animal data set comprises 76 metazoan taxa, 2 choanoflagellate outgroups, 212 genes, and 424 partitions representing first and second codon positions (Cannon et al. 2016). The tree reconstructed from all of the partitions (*T*_all_) is identical to the trees reconstructed from the 381 partitions that pass the MaxSymTest (*T*_pass_), the partitions that fail the MaxSymTest (*T*_pass_, 43 partitions), and the tree shown in the original paper from both DNA and amino acid data (Cannon et al. 2016), which places Xenacoelomorpha as the sister group of Nephrozoa (Deuterostomia and Protostomia) with 100% bootstrap support (supplementary extended figs. 1–3, Supplementary Material online).

## Discussion

In this article, we show that model violation is prevalent and has a strong impact on tree reconstruction in many phylogenetic data sets. This impact varies substantially between different data sets and different types of partitions. The trees inferred from different groups of partitions from the same data set often have topologies that are biologically and statistically significantly different.

Our results show great heterogeneity in the extent of model violation among different data sets and partitions. This is demonstrated by the varying proportion of partitions that failed the matched-pairs tests of homogeneity in each data set and in each genomic context (codon position, rRNA, tRNA, UCE, or other) and type of genome (nuclear, mitochondrial, plastid, and virus). Model violations are most frequently observed in the third codon positions for viral, mitochondrial and nuclear genomes, and intergenic spacers in plastid sequences. Yet, our results affirm that non-SRH evolution is far from constrained to these genomic regions. For example, in a data set of placental mammals, of the 22 partitions that failed the MaxSymTest, only 11 are third codon positions. The tree inferred from the partitions that show significant violation of the SRH conditions (*T*_fail_) differs in its topology from the tree inferred from the partitions that do not show significant violation of the SRH conditions (*T*_pass_) with respect to the interordinal relationships in Laurasiatheria (fig. 3). The tree inferred from partitions that violate the SRH conditions (*T*_fail_) is consistent with the results from the original paper in that it places Perissodactyla as a sister group to Carnivora + Pholidota (Lartillot and Delsuc 2012). However, other studies using ML analysis show Perissodactyla to be a sister group to Cetartiodactyla (Graur et al. 1997; Murphy et al. 2001; Tsagkogeorga et al. 2013; Liu et al. 2017), which is also the relationship we find in this study with the tree inferred from partitions that do not show significant violation of the SRH assumptions.

Examining the results of the two other tests (MaxSymTest_mar_ and MaxSymTest_int_) we noticed that all the partitions that failed the MaxSymTest also failed the MaxSymTest_mar_, suggesting that those partitions are violating the models mainly due to nonstationarity. Based on this observation, GC content may drive the differences between the trees inferred from all partitions and those inferred from partitions that failed neither MaxSymTest nor MaxSymTest_mar_. Trees with partitions that violate the models tend to group together clades with similar GC content (e.g., as in Betancur-r et al. 2013). However, it is hard to discern any clear evidence for this from examining the GC content of the clades (fig. 3). Yet, our results show that all the clades in the partitions that failed the MaxSymTest have on an average a higher GC content (fig. 3).

The results of our study also provide some insight into the likely cause of model violation in the data sets we examined. Figure 2 shows that violation of marginal symmetry (assessed with MaxSymTest_mar_) was much more common than violation of internal symmetry (assessed with MaxSymTest_int_). This suggests that nonstationarity, which is associated with marginal symmetry, is likely a more common cause of systematic bias than nonhomogeneity in the data sets that we examined (see also Jayaswal et al. 2005; Ababneh et al. 2006; Song et al. 2010). Yet, the difference between the proportion of partitions that failed the MaxSymTest_mar_ and the proportion of partitions that failed the MaxSymTest_int_ could also be due to the higher power of the MaxSymTest_mar_. Either way, this result hints that the development and application of nonstationary models (Yang 1994; Roberts and Yang 1995; Yap and Speed 2005) may be an important avenue toward reducing systematic bias in future analyses. Moreover, our results show a clear preference for simple substitution models with a single transition/transversion ratio over more complex models such as general time reversible. This suggests that developing nonstationary models with a single parameter for the transition/transversion ratio might be sufficient to reduce systematic bias in phylogenetic analysis.

One limitation of using the tests that we propose in this article is that their power will be limited if there are few differences between the sequences being examined. Indeed, our analyses show that in our representative sample of >3,500 partitions from published data sets, roughly ∼25% of the variance in whether a partition passes or fails a given test can be attributed to the number of observed differences between the sequences. Nevertheless, this implies that the remaining ∼75% of the variance in whether a partition passes or fails a test could be attributable to other processes, such as variation in the extent of model violation among partitions. This suggests that we should be cautiously optimistic: although a lack of power on small or slowly evolving partitions may induce some false negatives (i.e., failures to identify partitions that have evolved under non-SRH conditions), the tests we propose still have significant power to identify partitions that show the evidence of model violation. It is possible that removing such partitions from phylogenetic analyses may improve the accuracy of results by reducing the overall burden of model violation on the inference of the tree topology. We hope that our implementation of these tests in the user-friendly software IQ-TREE will allow empirical phylogeneticists to continue to explore whether this is the case.

## Supplementary Material

Supplementary data are available at *Genome Biology and Evolution* online.

## Supplementary Material

evz193_Supplementary_Data
