# Supplementary material for: The Prevalence and Impact of Model Violations in Phylogenetic Analysis
Source: Genome Biol Evol. 2019 Sep 19;11(12):3341–52. doi: 10.1093/gbe/evz193 (PMC6893154; doi:10.1093/gbe/evz193)
Supplement: evz193_Supplementary_Data [file evz193_supplementary_data.zip › Extended_Figures.docx]

**Extended Figure 1| ML topology of Cannon_2016 dataset inferred from all 424 partitions.**

**Extended Figure 2| ML topology of Cannon_2016 dataset inferred from all 281 partitions that passed the MaxSymTest.**

**Extended Figure 3| ML topology of Cannon_2016 dataset inferred from all 143 partitions that failed the MaxSymTest.**

**Extended Figure 4| ML topology of Lartillot_2012 dataset inferred from all 51 partitions.**

**Extended Figure 5| ML topology of Lartillot_2012 dataset inferred from all 29 partitions that passed the MaxSymTest.**

**Extended Figure 6| ML topology of Lartillot_2012 dataset inferred from all 22 partitions that failed the MaxSymTest.**


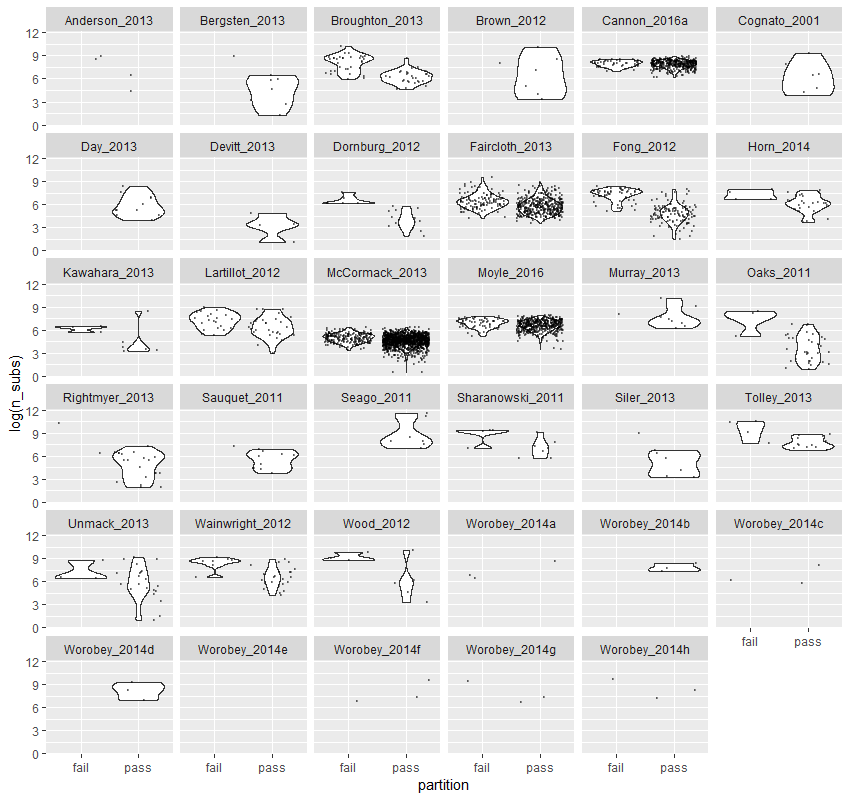


**Extended Figure 7| The number of substitution in partitions that failed or passed the MaxSymTest for each dataset.**


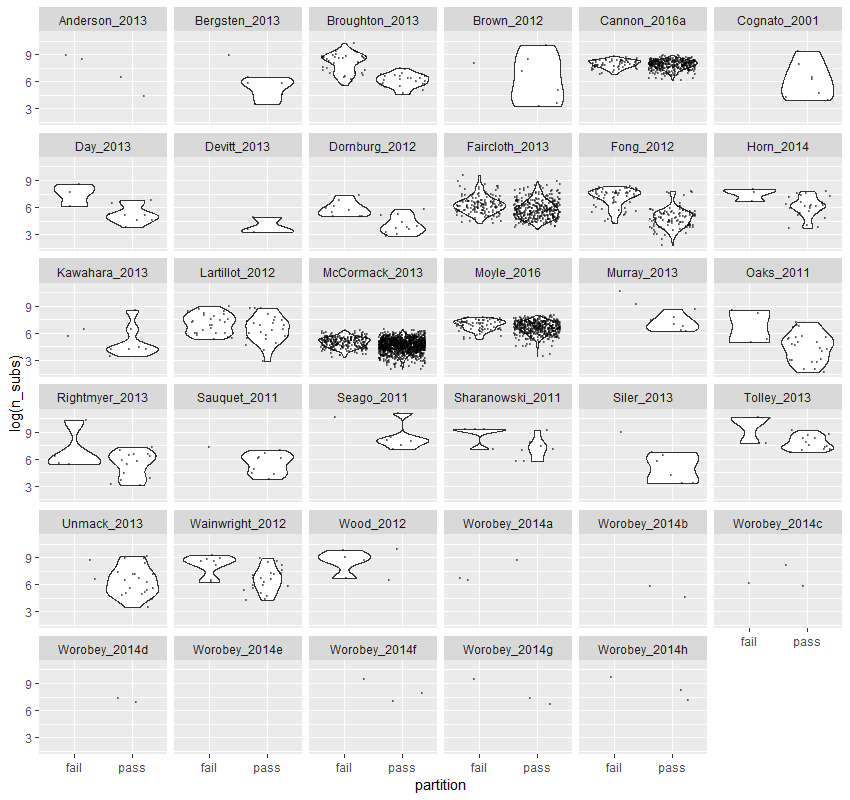


**Extended Figure 8| The number of substitution in partitions that failed or passed the MaxSymTest_mar_ for each dataset.**


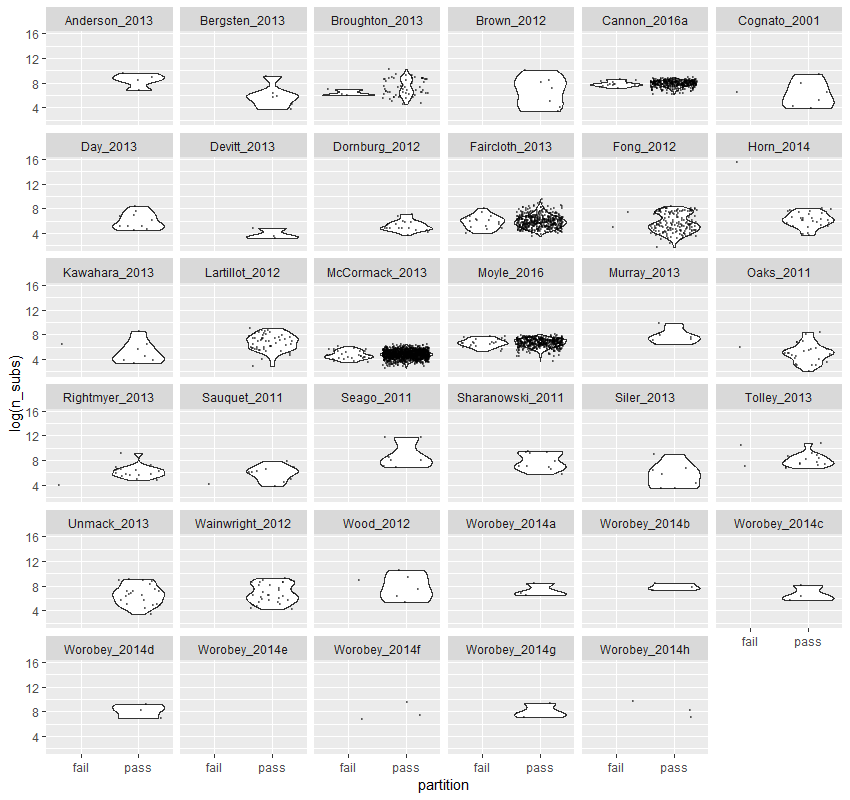


**Extended Figure 9| The number of substitution in partitions that failed or passed the MaxSymTest_int_ for each dataset.**
