# Supplementary material for: The Prevalence and Impact of Model Violations in Phylogenetic Analysis
Source: Genome Biol Evol. 2019 Sep 19;11(12):3341–52. doi: 10.1093/gbe/evz193 (PMC6893154; doi:10.1093/gbe/evz193)
Supplement: evz193_Supplementary_Data [file evz193_supplementary_data.zip › Extended_Tables.docx]

**Extended Table 1| best-fitting model by ModelFinder and number of partitions that got each model as the best-fit model**. Finding the best-fitting model (which minimize BIC score) for each one of the partitions.

| **Substitution model** | **#partitions with best-fit model** | **Nucleotide frequencies** |
| --- | --- | --- |
| K80 | 667 | Equal |
| HKY | 563 | Unequal |
| TPM2u | 297 | Unequal |
| TPM3u | 291 | Unequal |
| GTR | 272 | Unequal |
| TVM | 234 | Unequal |
| TIM3 | 164 | Unequal |
| TIM2 | 124 | Unequal |
| TN | 110 | Unequal |
| TVMe | 106 | Equal |
| TNe | 98 | Equal |
| TIM3e | 84 | Equal |
| K81 | 83 | Equal |
| K81u | 82 | Unequal |
| TIM2e | 80 | Equal |
| TPM3 | 60 | Equal |
| TPM2 | 54 | Equal |
| SYM | 53 | Equal |
| TIM | 41 | Unequal |
| JC | 32 | Equal |
| F81 | 21 | Unequal |
| TIMe | 16 | Equal |

**Extended Table 2| best-fitting model by ModelFinder for the partitions that passed each one of the three max-value tests.**

| **MaxSymTest** | |  | **MaxSymTest_mar_** | |  | **MaxSymTest_int_** | |
| --- | --- | --- | --- | --- | --- | --- | --- |
| model | #partitions |  | model | #partitions |  | model | #partitions |
| K80 | 548 |  | K80 | 503 |  | K80 | 622 |
| HKY | 514 |  | HKY | 489 |  | HKY | 522 |
| GTR | 256 |  | GTR | 238 |  | TPM3u | 287 |
| TPM2u | 243 |  | TPM3u | 236 |  | TPM2u | 287 |
| TPM3u | 239 |  | TPM2u | 232 |  | GTR | 266 |
| TVM | 177 |  | TVM | 163 |  | TVM | 224 |
| TIM3 | 135 |  | TIM3 | 138 |  | TIM3 | 151 |
| TIM2 | 102 |  | TIM2 | 90 |  | TIM2 | 123 |
| TN | 95 |  | TN | 84 |  | TVMe | 100 |
| TNe | 76 |  | TNe | 76 |  | TN | 96 |
| K81u | 66 |  | K81 | 63 |  | TNe | 91 |
| TPM3 | 65 |  | TIM3e | 61 |  | K81 | 81 |
| TVMe | 65 |  | TVMe | 59 |  | TIM3e | 77 |
| TIM3e | 64 |  | TPM3 | 56 |  | K81u | 75 |
| K81 | 64 |  | K81u | 55 |  | TIM2e | 74 |
| TIM2e | 56 |  | TIM2e | 51 |  | TPM3 | 57 |
| TPM2 | 49 |  | TPM2 | 42 |  | SYM | 51 |
| TIM | 34 |  | SYM | 33 |  | TPM2 | 51 |
| JC | 34 |  | TIM | 32 |  | TIM | 39 |
| SYM | 33 |  | JC | 29 |  | JC | 28 |
| F81 | 20 |  | F81 | 18 |  | TIMe | 17 |
| TIMe | 12 |  | TIMe | 13 |  | F81 | 15 |

**Exended Table 3| best-fitting model by ModelFinder for the partitions that failed each one of the three max-value tests.**

| **MaxSymTest** | |  | **MaxSymTest_mar_** | |  | **MaxSymTest_int_** | |
| --- | --- | --- | --- | --- | --- | --- | --- |
| model | #partitions |  | model | #partitions |  | model | #partitions |
| K80 | 128 |  | K80 | 157 |  | HKY | 22 |
| HKY | 59 |  | HKY | 74 |  | K80 | 14 |
| TVM | 48 |  | TVM | 68 |  | TVM | 9 |
| TPM2u | 46 |  | TPM2u | 52 |  | GTR | 8 |
| TPM3u | 38 |  | TPM3u | 51 |  | TIM3 | 8 |
| TVMe | 33 |  | GTR | 41 |  | TN | 7 |
| GTR | 27 |  | TVMe | 36 |  | TPM3u | 5 |
| TIM2e | 25 |  | TIM2 | 33 |  | TPM2 | 4 |
| TNe | 21 |  | TIM2e | 27 |  | TVMe | 4 |
| TIM3 | 21 |  | TNe | 23 |  | TIM2 | 4 |
| TIM2 | 21 |  | TIM3e | 21 |  | SYM | 3 |
| K81 | 20 |  | TN | 21 |  | JC | 3 |
| TN | 18 |  | TIM3 | 21 |  | TIM2e | 3 |
| TIM3e | 14 |  | K81u | 21 |  | TPM3 | 2 |
| SYM | 13 |  | K81 | 21 |  | TIM | 1 |
| K81u | 12 |  | SYM | 17 |  | TIM3e | 1 |
| TIM | 7 |  | TPM3 | 15 |  | K81u | 1 |
| TPM2 | 7 |  | TPM2 | 10 |  | TPM2u | 1 |
| TIMe | 6 |  | TIM | 8 |  | K81 | 1 |
| F81 | 2 |  | TIMe | 6 |  | SYM | 0 |
| JC | 0 |  | JC | 3 |  | F81 | 0 |
| TIMe | 0 |  | F81 | 2 |  | TIMe | 0 |

**Extended Table 4| The quartet distances between the three trees (T_all_, T_pass_, T_fail_) in MaxSymTest, MaxSymTest_mar_, and MaxSymTest_int_.**

|  | Dataset | T_all-fail_ | T_all-pass_ | T_fail-pass_ |
| --- | --- | --- | --- | --- |
| MaxSymTest | Anderson_2013 | 81372 | 4440183 | 4505628 |
|  | Bergsten_2013 | 25492 | 3902 | 27430 |
|  | Broughton_2013 | 35360 | 3738 | 39098 |
|  | Cannon_2016a | 20809 | 5746 | 26555 |
|  | Dornburg_2012 | 2373 | 6992 | 9365 |
|  | Faircloth_2013 | 442 | 0 | 442 |
|  | Horn_2014 | 1180177 | 975250 | 1823719 |
|  | Kawahara_2013 | 95727 | 38539 | 132150 |
|  | Lartillot_2012 | 303589 | 18326 | 297248 |
|  | McCormack_2013 | 10195 | 1243 | 10749 |
|  | Moyle_2016 | 78998 | 3031 | 82029 |
|  | Oaks_2011 | 68452 | 4142 | 72582 |
|  | Rightmyer_2013 | 441077 | 183468 | 568615 |
|  | Siler_2013 | 29961 | 11064 | 32949 |
|  | Wainwright_2012 | 3897669 | 1546897 | 5368384 |
|  | Wood_2012 | 539 | 13135 | 13076 |
|  | Worobey_2014a | 2699881 | 336202 | 2823981 |
|  | Worobey_2014c | 627673 | 8616 | 631824 |
|  | Worobey_2014e | 12707531 | 145471345 | 148579172 |
|  | Worobey_2014f | 160855578 | 4754274 | 162732984 |
|  | Worobey_2014g | 428217 | 22909429 | 23329085 |
|  | Worobey_2014h | 248010 | 43931488 | 44066427 |
|  |  |  |  |  |
| MaxSymTest_mar_ | Anderson_2013 | 130354 | 2284760 | 2294028 |
|  | Bergsten_2013 | 25492 | 8061 | 28962 |
|  | Broughton_2013 | 35360 | 3680 | 39040 |
|  | Cannon_2016a | 24582 | 3163 | 27745 |
|  | Day_2013 | 1458532 | 3555949 | 4668546 |
|  | Dornburg_2012 | 4052 | 16758 | 18728 |
|  | Faircloth_2013 | 442 | 0 | 442 |
|  | Horn_2014 | 1337822 | 364531 | 1365852 |
|  | Kawahara_2013 | 123280 | 15164 | 129332 |
|  | Lartillot_2012 | 21740 | 24730 | 43494 |
|  | McCormack_2013 | 6156 | 2674 | 7154 |
|  | Moyle_2016 | 90698 | 3031 | 93729 |
|  | Oaks_2011 | 83990 | 4004 | 87402 |
|  | Rightmyer_2013 | 426883 | 355486 | 595726 |
|  | Siler_2013 | 30018 | 10778 | 32835 |
|  | Wainwright_2012 | 4291174 | 2964931 | 5995967 |
|  | Wood_2012 | 58 | 11167 | 11223 |
|  | Worobey_2014a | 2688767 | 336156 | 2796992 |
|  | Worobey_2014b | 4846696 | 178703912 | 179250141 |
|  | Worobey_2014c | 268995 | 8583 | 275550 |
|  | Worobey_2014d | 21784654 | 92744994 | 78398319 |
|  | Worobey_2014e | 22662896 | 159964092 | 170213975 |
|  | Worobey_2014f | 3293336 | 14028740 | 17125648 |
|  | Worobey_2014g | 428217 | 22806396 | 23226052 |
|  | Worobey_2014h | 7666956 | 24536879 | 31423451 |
|  |  |  |  |  |
| MaxSymTest_int_ | Cannon_2016a | 221942 | 1323 | 223265 |
|  | Cognato_2001 | 1769 | 1728 | 41 |
|  | Faircloth_2013 | 412 | 0 | 412 |
|  | McCormack_2013 | 14090 | 687 | 14063 |
|  | Moyle_2016 | 75966 | 26979 | 102426 |
|  | Wood_2012 | 5867 | 3831 | 8679 |
|  | Worobey_2014f | 134222118 | 2786430 | 135193257 |
|  | Worobey_2014h | 7401114 | 22606490 | 27833436 |

**Extended Table 5| Number datasets that contain loci from the different types of genomes and the number of partitions from each type of genome.**

| **Genome type** | **#datasets** | **#genes** | **# partitions** |
| --- | --- | --- | --- |
| Mitochondria | 18 | 30 | 105 |
| Nuclear | 25 | 352 | 3419 |
| Plastid | 2 | 6 | 24 |
| Virus | 8 | 8 | 24 |
